# Serum Creatine Kinase as a Biomarker to Predict Wooden Breast *in vivo* for Chicken Breeding

**DOI:** 10.3389/fphys.2021.711711

**Published:** 2021-08-09

**Authors:** Fuli Kong, Guiping Zhao, Zhengxiao He, Jiahong Sun, Xicai Wang, Dawei Liu, Dan Zhu, Ranran Liu, Jie Wen

**Affiliations:** ^1^Institute of Animal Sciences, Chinese Academy of Agricultural Sciences, Beijing, China; ^2^State Key Laboratory of Animal Nutrition, Key Laboratory of Animal (Poultry), Genetics Breeding and Reproduction, Ministry of Agriculture, Beijing, China; ^3^Foshan Gaoming Xinguang Agricultural and Animal Industrials Corporation, Foshan, China

**Keywords:** creatine kinase, biomarker, predict, wooden breast, *in vivo*, chicken breeding

## Abstract

The present study aimed to find a blood marker for the prediction of wooden breast (WB) in live broiler to assist the genetic selection of fast-growing chickens. The experiments were carried out with two chicken flocks: 250 male broilers in flock 1 and 100 male and female broilers in flock 2. Both flocks were slaughtered and measured. The breast filets were assessed by combining subjective scoring and compression force at 28 (flock 1 only) and 42 days of age. The enzyme activity in serum and breast tissue (flock 1 only) of normal and affected groups was tested. The results showed that the compression force was significantly different between the normal and affected groups at 28 and 42 days of age (*P* < 0.001), and it increased significantly with rising WB and WS scores. The serum creatine kinase (CK) value increased significantly with rising compression force at 42 days of age (*P* < 0.001). The serum CK positively correlated with compression force (*r* = 0.608; *P* < 0.001) and the linear regression equation (serum CK = 0.9960^∗^compression force + 1.884) was established for the line studied. The association between serum CK and compression force is consistent between flocks 1 and 2. In conclusion, our study confirmed that compression force could be the quantitative indicator to differentiate breast filets and found that serum CK could be a candidate biomarker to predict WB in live broilers and assist genetic selection in broiler breeding.

## Introduction

The past five decades have witnessed an increase in consumer preference for chicken meat. To satisfy the still-growing demand for poultry meat, poultry industry experts have implemented different methods to increase the growth rate, feed efficiency, and muscle yield of broilers. However, artificial selection for the traits above in meat-type chickens has been associated with certain drawbacks, such as muscle fibers and meat quality alterations (Petracci et al., 2013; Velleman and Clark, 2015). To be specific, wooden breast (WB) features out-bulging and pale areas of hardened consistency and the distinctive traits of white striping (WS) is the presence of white striations parallel to the direction of muscle fibers (Kuttappan et al., 2012a, 2013b; Sihvo et al., 2014). WB and WS can appear together or individually and they are emerging globally in recent years. These abnormal breast filets can be visually detected at processing sites and lead to downgrades, as well as during packaging and marketing, and could contribute to consumer rejection. The problem is worsening and could hinder the advancement of the poultry industry (Zuidhof et al., 2014; Tallentire et al., 2018).

Researchers and breeding companies have studied and explored the contribution of genetic, nutrition, environmental, and management factors associated with the occurrence of WS and WB myopathies. Different dietary restriction schemes or some feed additives have only mild efficacy in decreasing the incidence of WS or WB (Bodle et al., 2018; Meloche et al., 2018a,b; Petracci et al., 2019a, b). The environmental enrichment does not appear to be a risk factor for WB (Pedersen et al., 2020). Furthermore, genetic selection has been found to have a significant effect on breast quality (Kuttappan et al., 2013a). In this regard, it was also reported that the heritability of WB was *h*^2^ < 0.1, WS was *h*^2^ ≤ 0.338 (Bailey et al., 2015), and in the pHu + line WS was *h*^2^ = 0.65 (Alnahhas et al., 2016). Moreover, Pampouille et al. (2018) explained that there is no major gene responsible for the occurrence of WS, but rather a polygenic inheritance exists. Lake et al. identified candidate genes for WB and WS in commercial broiler chickens, such as *KCNQ1* and *LSP1* (Lake et al., 2021). According to previous research, poultry breeding could be an important part of eliminating broiler meat abnormalities in the future. Before key genes and mutations that control WS and WB are resolved, live broiler breeder selection may be an effective way to reduce the occurrence of abnormal breast filets.

Kawasaki et al. (2016) reported the ability of wing lifting as a diagnostic method for WB broilers. Nevertheless, screening broilers only by appearance may neglect some less severe individuals. It is also reported breast echogenicity can be an additional tool to early detect alterations related to WB, and the correlation between WB and echogenicity was 0.430 at 42 days (Simoes et al., 2020). There is still need for quantitative and convenient methods to assess breast meat defects in live broilers. It is known that the damage that occurs in muscle tissue could be reflected in plasma or serum biochemical profiles. It was reported that the damage of muscle tissue could disrupt the integrity of the sarcolemma resulting in the leaking of various enzymes, such as creatine kinase (CK) and lactate dehydrogenase (LDH), into the plasma or serum (Hoffman and Solter, 2008). Moreover, in turkeys, it was also demonstrated that individuals with plasma CK levels above 1,000 units/ml could be considered susceptible to deep pectoral myopathy (Siller, 1985). We could not find a report that focuses on serum enzyme activity and the degree of WB. The purpose of this study is to assess whether different degrees of WB can be reflected in serum enzyme levels and try to find the boundary value to screen live broilers with breast meat defects in broiler breeding.

## Materials and Methods

### Broilers Husbandry and Sample Collection

All the chickens in this study were obtained from a 9th generation (G9) fast-growing white-feathered line B chickens and produced by the Xinguang Agricultural and Animal Industrials Co., Ltd. (Mile, China). The broilers of flock 1 were raised by the company farm. Chickens in flock 2 were raised by IAS in the CAAS Experimental Base. All Broilers were raised in stair-step cages with individual pens under the same recommended environmental and nutritional conditions (Feeding Standard of Chickens, China, NY 33-2004). Broilers were free access to feed and water and the composition of ingredients used in the diets is shown in Table 1.

**TABLE 1:** Ingredient composition of diets fed to Line B broilers from 1 to 42 days of age.

**Item**	**Pre-Starter 1–7 days**	**Starter 8–20 days**	**Grower 21–34 days**	**Finisher 35–42 days**
**Ingredients, %**				
Corn	54.8	57.5	58.5	62
Soybean meal	38.22	35	32.33	28.53
Soybean oil	4.5	5	5.2	5.5
Limestone	0	0	1.95	1.95
Dicalcium phosphate	1.72	1.74	1.35	1.35
Salt	0.3	0.3	0.3	0.3
DL-Met	0.26	0.26	0.16	0.16
Premix compound	0.2	0.2	0.2	0.2
**Nutrient composition, %**				
ME, MJ/kg	12.78	12.99	12.94	13.13
CP	22.1	20.9	19	18
Ca	0.5	0.5	0.57	0.55
Nonphytate P	0.4	0.4	0.31	0.3
Lys	1.1	1.1	1	1
Met	0.6	0.6	0.42	0.42

*For starter diet, premix compound provided per kilogram of diet: vitamin A 12,500 IU; vitamin D 3,750 IU; vitamin E 33 IU; vitamin K 2.5 mg; thiamin 2.5 mg; riboflavin, 10.0 mg; vitamin B6, 2.5 mg; vitamin B12, 0.015 mg; calcium pantothenate, 15 mg; niacin 32.5 mg; folic acid 1.25 mg; biotin 0.125 mg; choline 700 mg; Cu 8 mg; Mn 100 mg; Fe 80 mg; Zn 60 mg; I 0.35 mg; Se 0.15 mg. For grower diet, premix compound provided per kilogram of diet: vitamin A 10,000 IU; vitamin D3,300 IU; vitamin E 20 IU; vitamin K 2.0 mg; thiamin 2.0 mg; riboflavin 8.0 mg; vitamin B6 2.0 mg; vitamin B12 0.01 mg; calcium pantothenate 12.5 mg; niacin 26.0 mg; folic acid 1.0 mg; biotin 0.10 mg; choline 500 mg; Cu 8 mg; Mn 80 mg; Zn 40 mg; Fe 80 mg; I 0.35 mg; Se 0.15 mg. For finish diet, premix compound provided per kilogram of diet: vitamin A 9,500 IU; vitamin D 3,000 IU; vitamin E 16 IU; vitamin K 2.0 mg; thiamin 1.8 mg; riboflavin 6.4 mg; vitamin B6 2.0 mg; vitamin B12 0.01 mg; calcium pantothenate 10 mg; niacin 26.0 mg; folic acid 1.0 mg; biotin 0.10 mg; choline 500 mg; Cu 6 mg; Mn 60 mg; Zn 30 mg; Fe 80 mg; I 0.35 mg; Se 0.15 mg.*

Broilers of flock 1 were fasted for 12 h and individually weighed at 28 and 42 days of age. The average weights of the 100 broilers at 28 and 150 broilers 42 days of age were determined, respectively, in the flock 1. The blood samples were collected from the wing vein and centrifuged (2,500 RPM for 10 min) to obtain serum and stored at −80°C until analyses of enzyme activity. All the broilers were electrically stunned, bled for 3 min, and scalded at 60°C for 45 s to de-feather. The right breasts were snap-frozen immediately in liquid nitrogen then stored at −80°C for subsequent analyses. The left breasts were deboned by a small group of trained personnel (6–8 people) to avoid any filet weight difference and stored in 4°C for 3 h before scoring. Broilers of flock 2 were also collected blood and left breast samples at 42 days, and the procedure was the same as for flock 1.

### Wooden Breast and White Striping Scores

All the deboned left filets were submitted to trained personnel (2–3 people) to provide WB and WS scores. The WB scores were as described by Tijare et al. (2016) and included the absence of WB (normal breast score 0), mild hardening in the upper part of the filet (score 1), moderate hardening in the upper and/or lower part of the filet (score 2), severe hardening (score 3) and severe hardening with hemorrhagic lesions, increased volume and presence of yellow fluid (score > 3). The WS scores included no distinct white line (normal breast score 0), moderate WS (small white lines, generally <1 mm thick, but visible on the filet surface score 1), severe WS (white lines 1–2 mm thick, very visible on the filet surface, score 2), and extreme severe WS (thick white bands > 2 mm thickness, covering almost the entire surface of filet, score 3; Kuttappan et al., 2012b).

### Compression Force Measurement

The left filets were used for compression force analysis according to the method reported by the Sun et al. (2018). Filets were compressed to 20% on different cranial regions using a 6-mm flat probe on a TA.XT Plus Texture Analyzer (Stable Micro Systems Ltd., Godalming, United Kingdom). The trigger force was set at 5 g, probe height was set at 55 mm (higher than the thickest filet sample); pre- and post-probe speeds were both 10 mm/s and the test speed of the probe was 5 mm/s. All the breast filets were measured three times at different positions of the filet cranial region, and the average compression force values were used for statistical analysis.

### Enzyme Activity in Serum and Breast Tissue

The serum stored at −80°C were used to estimate the activities of CK, LDH, aspartate transaminase (AST), and alanine transaminase (ALT) and the right breast tissues were used to estimate the activities of CK and LDH. The brief procedures are as below: 100 mg breast tissue and 0.9 ml 0.9% NaCl solution was put in the tube and smashed for 1–2 min with tissue grinder, then centrifuged at 2,500 *g* for 10 min at 4°C to obtain the supernatant. Total protein concentrations in the supernatant were measured using a BCA protein assay kit (Nanjing Jiancheng Bioengineering Institute, Nanjing, China). The enzyme activity in breast supernatant and serum were determined using the detection kit (Nanjing Jiancheng Bioengineering Institute, Nanjing, China). All the procedures were carried out according to the manufacturers’ instructions. Enzyme activity in breast tissue and serum were expressed as units per milligram of protein and units per milliliter, respectively.

### Statistical Analyses

In this study, the dietary treatment and all management factors were the same to avoid any confounding effects. Each chicken was an individual experimental unit. All results were presented as mean and standard deviation and analyzed by GraphPad Prism 8 software (GraphPad Software, Inc. San Diego, CA, United States). One-way analysis of variance was used to compare the mean differences among the different groups. *P* < 0.05 or 0.01 was assigned as a significance level.

## Results

### Assessment of Breast Filet and Identification of Candidate Biomarkers

Bodyweight, filet weight and the scores of the WB and WS at 28 and 42 days of age are shown in Table 2. Despite the similar bodyweight of the two groups, the filet weight of the mild group was significantly higher than the normal group at 28 days of age (*P* < 0.01). There were no severe WS and WB filets in this study and more than half of the affected breast filets appeared as WS and WB together at 42 days of age. The bodyweight and filet weight of the moderate WB group were significantly higher than the other two groups (*P* < 0.05), and there was no significant difference according to the WS score (*P* > 0.05). Spearman’s correlation coefficients between the WB and WS scores, bodyweight, and filet weights are presented in Table 3.

**TABLE 2:** Bodyweight, filet weight, and wooden breast and white striping scores at 28 and 42 days of age in flock 1.

**Group**	**Score**	**Body weight (g) (mean ± SD)**	**Filet weight (g) (mean ± SD)**	***N***
**28 days**				
NORM	WS + WB = 0	1,288 ± 6	120 ± 3^a^	10
MILD	WB = 1	1,274 ± 29	134 ± 3^b^	10
*P*-value		ns	<0.01	
**42 days**				
WB score				
NORM	WB = 0	2,530 ± 138^b^	241 ± 19^b^	67
MILD	WB = 1	2,528 ± 121^b^	242 ± 45^b^	37
MOD	WB = 2	2,603 ± 175^c^	259 ± 25^c^	31
*P*-value		<0.05	<0.05	
WS score				
NORM	WS = 0	2,531 ± 151	241 ± 22	73
MOD	WS = 1	2,565 ± 137	250 ± 38	62
*P*-value		ns	ns	
WS and WB				
NORM	WS + WB = 0	2,520 ± 127^a^	236 ± 17^a^	39
MILD	WS + WB ≥ 1	2,536 ± 151^a^	245 ± 35^a^	77
MOD	WS + WB = 3	2,639 ± 123^b^	268 ± 15^b^	19
*P*-value		<0.01	<0.01	

*^*a,b*^Different letters indicate a significant difference (*P* < 0.01). ^*b,c*^Different letters indicate a significant difference (*P* < 0.05).*

**TABLE 3:** Spearman’s correlation coefficients between WB and WS scores, bodyweight, filet weight and compression force in flock 1.

**Category**	**WB**	**WS**	**WB + WS**	**Body weight**	**Filet weight**	**Compression force**
WB	1					
WS	0.138	1				
WB + WS	0.872**	0.606**	1			
Body weight	0.179*	0.119	0.203*	1		
Filet weight	0.224**	0.146	0.253**	0.500**	1	
Compression force	0.718**	0.336**	0.693**	0.094	0.228**	1

WB, wooden breast score; WS, white striping score; WS + WB, wooden breast score plus white striping score. **Significantly different (*P* < 0.01). *Significantly different (*P* < 0.05).

**FIGURE 1:**
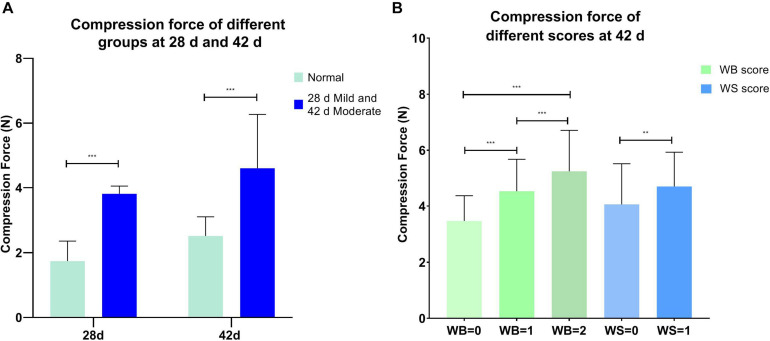
**(A)** Compression force in the normal and mild/moderate groups at 28 (*n* = 20 in total) and 42 days of age (*n* = 49 in total); **(B)** compression force of different wooden breast and white striping scores at 42 days of age (*n* = 135 in total). ***P < 0.001; **P < 0.01.

The compression force was significantly different between the normal and affected group at 28 and 42 days (*P* < 0.001, Figure 1A), and it increased significantly with the rising WB and WS scores (*P* < 0.01, Figure 1B) at 42 days. WB = 0 (normal group), WB = 1 (mild group), and WB = 2 (moderate group) corresponded to compression forces 3.18 ± 0.55 N, 4.62 ± 0.34 N, and 6.42 ± 0.95 N, respectively. WS = 0 (normal group) and WS = 1(moderate group) could be represented by compression force 4.06 ± 1.45 N and 4.70 ± 1.20 N. As shown in Table 3, compression force had a positive strong correlation (*P* < 0.001) with WB score (*r* = 0.718) and WB + WS score (*r* = 0.693). All the observations demonstrated that compression force could be the quantitative indicator to differentiate WB affected breast filets.

**FIGURE 2:**
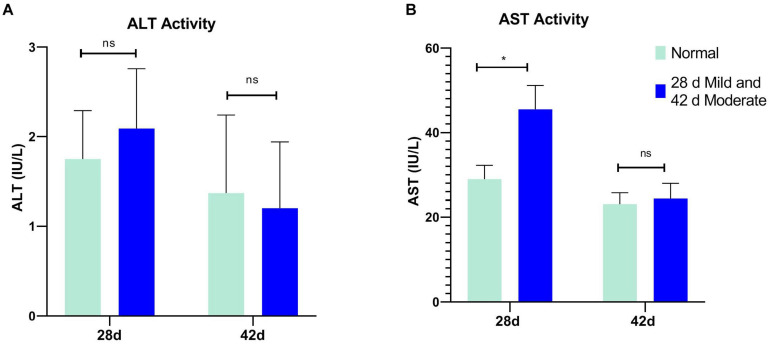
Serum alanine transaminase (ALT) and aspartate transaminase (AST) activity at 28 (*n* = 20 in total) and 42 days of age (*n* = 49 in total). **(A)** ALT activity; **(B)** AST activity. *P < 0.05; ns, *P* > 0.05.

**FIGURE 3:**
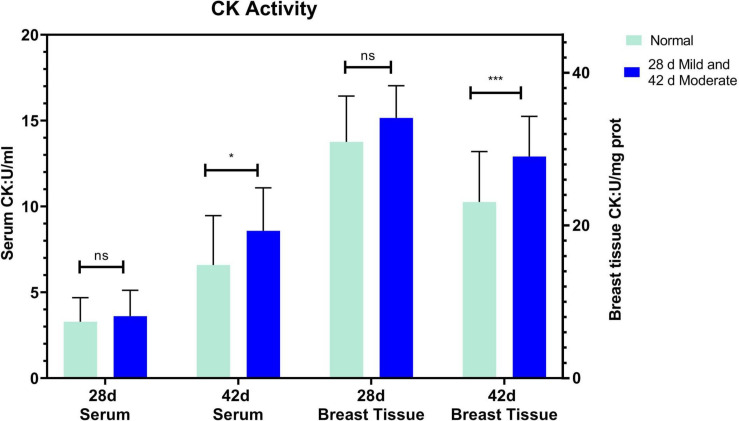
Creatine kinase (CK) activity in serum and breast tissue at 28 (*n* = 20 in total) and 42 days of age (*n* = 49 in total). ***P < 0.001; *P < 0.05; ns, *P* > 0.05.

**FIGURE 4:**
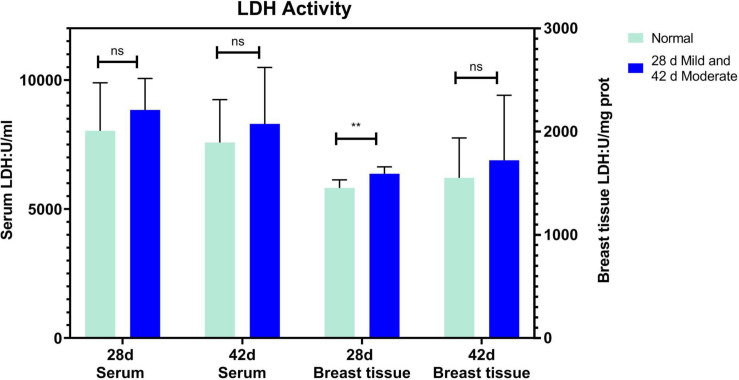
Lactate dehydrogenase activity in serum and breast tissue at 28 (*n = 20* in total) and 42 days of age (*n* = 49 in total). **P < 0.01; ns, *P* > 0.05.

The serum ALT of the normal and affected group had no significant differences at 28 and 42 days (*P* > 0.05, Figure 2A). The serum AST of the mild group was significantly higher than the normal group (*P* = 0.031) at 28 days but not at 42 days (*P* > 0.05, Figure 2B). CK activity in serum and breast tissue of the moderate group was remarkably higher than in the normal group at 42 days of age (*P* < 0.05, *P* < 0.001, Figure 3). The tendency of the two groups at 28 days was the same as at 42 days but did not show a significant difference (*P* > 0.05, Figure 3). The LDH activity of the mild and moderate group in serum was higher than in the normal group at 28 and 42 days, but there were no significant differences (*P* > 0.05, Figure 4). These results indicated that CK could be a potential blood marker to predict defects in the breast filet.

### Validation of Serum Creatine Kinase as a Biomarker

**FIGURE 5:**
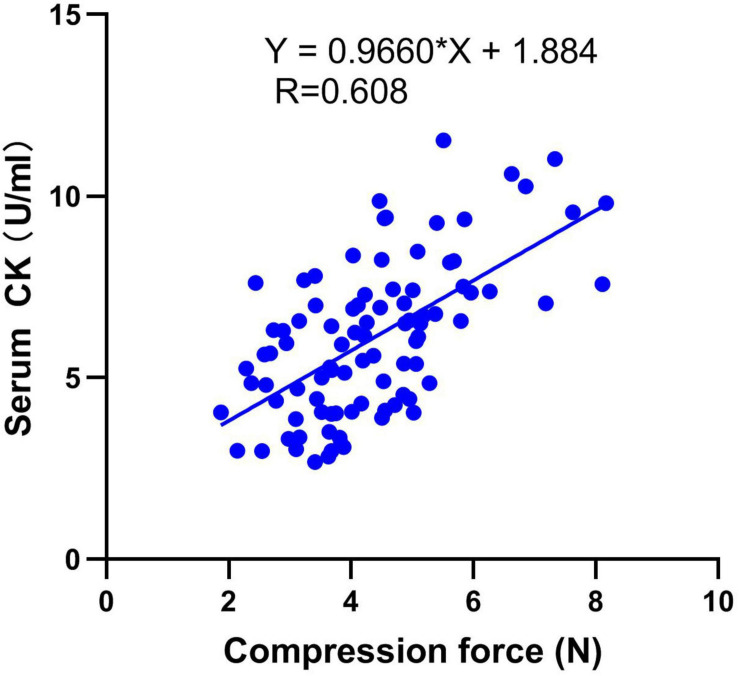
Correlation analysis between serum CK level and compression force (*r* = 0.608, *P* < 0.001). *n* = 92.

The above results demonstrate that compression force could be the quantitative indicator to differentiate breast filets. To verify the biomarker, serum was grouped by compression force as shown in Table 4. The results showed that the CK value increased significantly with compression force increasing at 42 days of age (*P* < 0.001). Correlation analysis between serum CK level and compression force is described in Figure 5. It shows that the serum CK level positively correlated with compression force (*r* = 0.608, *P* < 0.001). The equation of linear regression was described as serum CK = 0.9960^∗^compression force + 1.884.

**TABLE 4:** Serum creatine kinase (CK) level of different groups at 42 days of age in flock 1.

**Group**	**Compression force range (N)**	**Compression force (N)**	**Creatine kinase (U/ml)**	***N***
Normal	<4	3.2 ± 0.6^a^	4.8 ± 1.5^a^	39
Mild	4–5.5	4.6 ± 0.3^b^	6.3 ± 1.6^b^	36
Moderate	≥5.5	6.4 ± 1.0^c^	8.7 ± 1.6^c^	17
*P*-value		<0.001	<0.001	

*^*a,b,c*^Different letters indicate significant difference (*P* < 0.001).*

To verify the results, the flock 2 serum was divided into three groups according to the criteria set by flock 1. The results showed that serum CK value increased significantly with rising compression force (*P* < 0.05, Table 5).

**TABLE 5:** Serum CK level of different group at 42 days of age in flock 2.

**Group**	**Compression force range (N)**	**Compression force (N)**	**Creatine kinase (U/ml)**	***N***
Normal	<4	2.4 ± 0.9^a^	4.6 ± 1.3^d^	49
Mild	4–5.5	4.3 ± 0.2^b^	5.1 ± 1.5^e^	13
Moderate	≥5.5	6.2 ± 0.8^c^	6.4 ± 1.5^f^	8
*P*-value		<0.001	<0.05	

*^*a,b,c*^Different letters indicate significant difference (*P* < 0.001), ^*d,e,f*^different letters indicate significant difference (*P* < 0.05).*

## Discussion

Over the past decade, the increasing occurrence of WS and WB in commercial broilers has triggered considerable concern in the poultry industry. Previous studies suggested that the main reason for the myopathies might be linked to the intensive broiler selection scheme for massive breast yield (Kuttappan et al., 2012a, 2013a; Petracci et al., 2013; Malila et al., 2018). The bodyweight and breast filet weight of the WB affected broilers were significantly higher than that of normal broilers in the studied line (*P* < 0.05). Published data, as well as data from the present study, corroborate that the growth rate, especially the breast filet yield may be the origin of the myopathies. The rapid growth and inadequate vascularization in breast induced the hypoxic conditions, then kinds of response mechanisms such as energetic metabolism, inflammation, degeneration, and regeneration were modified or triggered. All these alterations were strictly related to the progression of myopathic disorders (Soglia et al., 2021). In addition, significant accumulation of lipofuscin was associated with the development of WB (Hasegawa et al., 2021). Fibrosis was also one of the main features of WB and this may be related to the activation of transforming growth factor-beta signaling and the dysregulation of the matrix metalloproteinases and tissue inhibitor of metalloproteinases system (Xing et al., 2021). Besides, Praud et al. (2020) suggested that a balance between *TGFB1* and *PPARG* would be essential for fibrosis or adiposis induction during the development of WB and WS. All the results suggested that there are several biological pathways, as well as response mechanisms involved in the development of WB.

Generally, breast meat defects are subjectively judged by prominent characteristics. WB was identified by assessing the filet hardness and WS was divided by the thickness of white stripes (Sihvo et al., 2014; Tijare et al., 2016). Dalgaard et al. (2018) reported a classification method of WB affected breast including the moisture content, resistance to compression, mobile water fraction, drip loss and cooking loss, as well as intramuscular and surface pH. This method removed the subjectiveness of the previous method, but need many laboratory tests. Thus, there is still need for an objective and fast method to quantify the meat defects. Previous research found that the compression test can be an objective and effective tool to identify and determine the severity of WB (Mudalal et al., 2015; Soglia et al., 2017; Sun et al., 2018; Campo et al., 2020). In this study, despite no severe WB and WS, compression force increased significantly with the increase in WB and WS scores. Different compression force ranges could present normal, mild, and moderate WB and WS groups. Moreover, compression force had a strong positive correlation (*P* < 0.001) with WB score (*r* = 0.718) and WB + WS score (*r* = 0.693). These results suggest that compression force could be an objective and quantitative index to differentiate the affected breast filets, especially for WB and when WB and WS appear together in breast meat.

It was reported that increasing levels of ALT, AST, CK, and LDH were associated with liver or muscle damage; and that a CK assay with blood samples was mainly a major marker of skeletal muscle injury (Hoffman and Solter, 2008; Lumeij, 2008). Kuttappan et al. (2013b) showed that broilers with severe WS (*P* < 0.05) had elevated serum levels of CK, ALT, AST, and LDH. Kawasaki et al. (2018) demonstrated that serum CK and AST values in most WB affected broilers were higher than those in normal broilers at 20 days of age. In addition, research by Meloche et al. (2018a, b)) demonstrated that plasma CK and LDH increased (*P* < 0.05) with increasing WB and WS scores. However, they believed that LDH may have more value as a predictor of myopathy. Serum CK was selected as an indicator to assess the effects of different feed schemes in reducing the occurrence of WS and WB (Simoes et al., 2020) and can be used as an independent factor for predicting amyotrophic lateral sclerosis in patients (Chen et al., 2021).

In this study, the liver damage was slight because there was no significant difference in ALT and AST at 42 d of age. This result indicated that most of the serum enzyme activity variation was derived from muscles or other organs but not the liver at 42 days. The AST level of the mild group was significantly higher than the normal group at 28 days. This alteration may relate to myocardial injury and functional compensation due to rapid weight gain with a high metabolic rate and more oxygen demand at 28 days of age. This result was in step with Takeshi Kawasaki et al. (2018). Changes in LDH serum levels did not respond to the degree of WB and WS which were not wholly consistent with previous reports (Meloche et al., 2018a,b). Different broiler lines and different degrees of WB and WS may contribute to the variations. Also, the LDH level in serum was higher than that of breast tissue and indicated that it may be inappropriate to take LDH as a predictor of WB and WS.

The serum CK value was about 10–25% of that in breast tissue, suggesting that the breast defects may make a great contribution to the serum CK. However, all the serum CK values increased with age but the moderate group was significantly higher than the normal group at 42 days (*P* < 0.05) and the tendency was the same at 28 days (*P* > 0.05). These indicated that despite serum CK value increasing with age, it mirrored the damage of the breast issue well and could be a candidate blood marker to predict filet defects at 42 days of age. Besides, the correlation between serum CK and compression force at 35 d or earlier deserves to study to screen the affected broilers as early as possible. Moreover, the results of the confirmatory experiment showed that the serum CK value increased significantly with compression force increasing in the two chicken flocks (*P* < 0.001). However, the compression force and CK value of flock 2 were lower than that of flock 1, which could be explained by different gender because the occurrence of WB and WS in male broilers was higher than that of female broilers (Trocino et al., 2015).

Most importantly, the serum CK positively correlated with compression force (*r* = 0.608, *P* < 0.001) and the equation of the linear regression was described as serum CK = 0.9960^∗^compression force + 1.884. Because different compression force ranges could present normal, mild, and moderate WB groups for specific line, the breast compression force of live broilers can be predicted and the WB broilers could be screened by serum CK detection. However, the incidence of WB and the production performance of their offspring should be analyzed when discarding broilers by this method, because keeping a good balance between high-quality breast and high yield is a great challenge for broiler breeding. Furthermore, to avoid adverse effects on breast yield, the genetic correlation between serum CK level and filet weight should be studied further in the next generation.

## Conclusion

In conclusion, this study confirmed that compression force could be the quantitative indicator to differentiate breast filets and found that serum CK could be an objective testing method for the prediction of compression force through the linear regression equation in broiler breeding. Different compression force ranges could represent normal or affected groups for specific line, so the WB affected broilers could be screened by serum CK detection.

## Data Availability Statement

The original contributions presented in the study are included in the article/supplementary material, further inquiries can be directed to the corresponding authors.

## Ethics Statement

Broiler experiments were conducted under the guidelines for experimental animals established by the Ministry of Science and Technology (Beijing, China). Ethical approval on animal survival was given by the animal welfare and ethics committee of Institute Animal Sciences (IAS, Beijing, China) and the Chinese Academy of Agricultural Sciences (CAAS, Beijing, China) with the following reference number: IAS2019-44.

## Author Contributions

FK collected all the data, performed the analyses, and drafted the first manuscript. GZ, ZH, and JS contributed to the data interpretation and manuscript revision. XW, DL, and DZ contributed to the chicken raising, sample and data collection. JW and RL designed the research and contributed to data collection, data analysis and interpretation, and revise the manuscript. All authors submitted comments on the draft and approved the final manuscript.

## Conflict of Interest

The authors declare that the research was conducted in the absence of any commercial or financial relationships that could be construed as a potential conflict of interest.

## Publisher’s Note

All claims expressed in this article are solely those of the authors and do not necessarily represent those of their affiliated organizations, or those of the publisher, the editors and the reviewers. Any product that may be evaluated in this article, or claim that may be made by its manufacturer, is not guaranteed or endorsed by the publisher.
